# Chocolate bar as an incentive did not increase response rate among physiotherapists: a randomised controlled trial

**DOI:** 10.1186/1756-0500-1-34

**Published:** 2008-06-24

**Authors:** Gro Jamtvedt, Sarah Rosenbaum, Kristin Thuve Dahm, Signe Flottorp

**Affiliations:** 1 Norwegian Knowledge Centre for the Health Services, PO Box 7004, St. Olavs plass, 0103 Oslo, Norway

## Abstract

**Background:**

The aim of this study was to assess the effect of a small incentive, a bar of dark chocolate, on response rate in a study of physiotherapy performance in patients with knee osteoarthritis.

**Findings:**

Norwegian physiotherapists from private practice were randomised in blocks to an intervention group (n = 1027) receiving a bar of dark chocolate together with a data-collection form, and a control group (n = 1027) that received the data-collection form only. The physiotherapists were asked to prospectively complete the data-collection form by reporting treatments provided to one patient with knee osteoarthritis through 12 treatment sessions. The outcome measure was response rate of completed forms.

Out of the 510 physiotherapists that responded, 280 had completed the data-collection form by the end of the study period. There was no difference between the chocolate and no-chocolate group in response rate of those who sent in completed forms. In the chocolate group, 142 (13.8%) returned completed forms compared to 138 (13.4%) in the control group, ARR = 0.4 (95% CI: -3.44 to 2.6).

**Conclusion:**

A bar of dark chocolate did not increase response rate in a prospective study of physiotherapy performance. Stronger incentives than chocolate seem to be necessary to increase the response rate among professionals who are asked to report about their practice.

**Trial Registration:**

Current Controlled Trials register: ISRCTN02397855

## Background

Non-response to postal questionnaires is a well known problem that can introduce bias in surveys and in epidemiological studies. Several ways of increasing response rate have been identified, and research has shown that the odds of response can double using monetary incentives [1]. Even small financial incentives are found to be effective in improving physician response [2]. Non-monetary incentives can also be effective, though should be handed out together with questionnaires rather than afterwards [1]. Interventions that trigger positive emotions, such as candy, have also been shown to have an effect on trial participants' willingness to solve tasks and to increase response rate among physicians [3-5].

Problems with non-response have been demonstrated in surveys of practice performance in health care [6,7]. The quality of research within this field could be improved by identifying ways to increase response. Therefore, while planning a prospective study of physiotherapy performance in Norway we decided to test the effect of a non-monetary incentive on response. To our knowledge no study has evaluated the effect of chocolate. Thus, the aim of this study was to assess the effect of a bar of dark chocolate on response rate in a study of physiotherapy performance in patients with knee osteoarthritis.

## Methods

**Figure 1:**
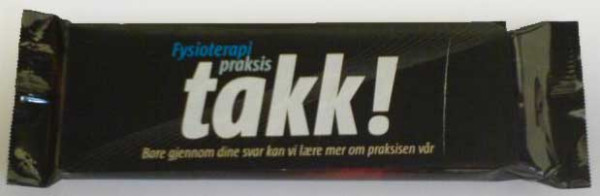
Bar of chocolate with sticker.

In May 2006 all Norwegian physiotherapists in private practice (n = 2798) were invited to participate in a prospective study measuring physiotherapy performance for knee osteoarthritis [8]. Based on feedback from the first invitation, 744 were considered not eligible, mainly because they did not treat patients with osteoarthritis. The remaining physiotherapists were randomly assigned to an intervention group (n = 1027) that received a bar of chocolate together with the data-collection form, and a control group (n = 1027) that received the data-collection form only. The physiotherapists were randomised in blocks of six by a computer generated table. We distributed the forms and chocolates by postal mail including a pre-paid return envelope. The chocolate bar consisted of 36 grams 70% cacao, wrapped in a specially designed sticker bearing survey logo and the text "Thank you for helping us to document physiotherapy practice", Figure 1.

The six-page long data-collection form was developed through several steps involving clinicians and experts. It was designed to prospectively report treatments provided to one patient with knee osteoarthritis through 12 treatment sessions. There were three sections including questions about physiotherapist and patient characteristics.

After the first mailing all physiotherapists were sent one follow-up reminder by mail and one by e-mail. All practices with more than five physiotherapists were also contacted by telephone. The study period spanned over nine months.

The proportion of completed data-collection forms (response rate) was the primary outcome.

By assuming a worst case response rate of 20% and with 1094 participants in each arm, the study had 80% power to detect a 5% increase in response rate in the chocolate group.

## Results

**Figure 2:**
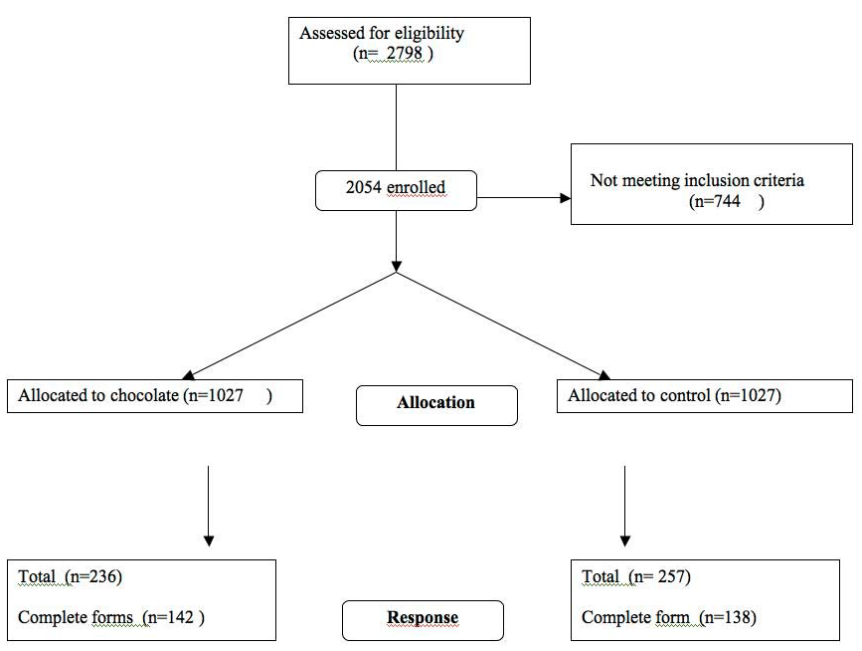
Flow diagram.

We received a response from 510 physiotherapists (236 in the chocolate group and 257 in the no-chocolate group). Some stated that they did not treat patients with knee osteoarthritis or they reported other reasons for not participating, such as not working in clinical practice or focusing on areas like neurology, child or mental health. Among the responders 280 had completed the data-collection form (Figure 2). Before the first reminder was sent out we had received 73 completed forms, 39 (3.8%) from the chocolate group and 34 (3.3%) from the no-chocolate group. By end of the study there was no difference between the chocolate and no-chocolate group in the number of completed forms, 142 (13.8%) in the chocolate group and 138 (13.4%) in the control group, ARR = 0.4 (95%-CI: -3.4 – 2.6).

## Discussion

In this study we evaluated the effect of a bar of dark chocolate on response rate in a prospective study of physiotherapy performance. The overall response rate was very low and the chocolate bar did not improve the number of completed data-collection forms. The findings are similar to the study by Halpern et al which found that mints did not influence response rate in a mailed questionnaire among physicians [5], and support findings from a systematic review on effects of incentives to improve response rates to physician surveys that concluded that token nonmonetary incentives were much less effective than even small financial incentives [2]. One explanation to our findings may be the time lapse between receiving the chocolate and performing the requested tasks, or the amount of work requested. The study required subjects to document treatment over a period of several weeks, and chocolate did not seem to have had a strong enough influence or one that lasted long enough to produce the desired effect. All physiotherapists were sent two reminders. These reminders may have prompted both groups to respond equally, cancelling out any effect of the chocolate.

The overall response rate was very low in this study although we tried to prevent non-response in different ways. We contacted participants before they received the questionnaire, the questionnaires were sent by first class post and stamped-return envelopes were provided and we sent two reminders [1,2]. It was professionally designed and kept as short as possible. However, if the use of short questionnaires reduces the accuracy of the measurement process, there are trade-offs between non-response and less precise measurement.

## Conclusion

There are many barriers for health professionals in reporting their practice behaviour. Adding one bar of chocolate did not seem to be a sufficiently strong incentive to increase the response rate.

## Competing interests

We enjoy eating chocolate, but still all authors declare that they have no competing interests.

## Authors' contributions

SR had the idea about the chocolate incentive. GJ wrote the protocol and designed the study performed the analysis and drafted the first version of the manuscript. KTD contributed to designing the study, entered data into SPSS and revised drafts of the manuscript. SR and SF contributed to the idea of the project and to design and analysis and revised drafts of the manuscript. All authors approved the final manuscript
